# The Use of Botulinum Toxin Type A in Esophageal Atresia Management: a Randomized, Controlled, Investigator-blinded Feasibility and Exploratory Trial in Piglets

**DOI:** 10.1038/s41598-026-59876-y

**Published:** 2026-06-30

**Authors:** Emma Svensson, Peter Zvara, Lars Hagander, Lars Rasmussen, Niels Qvist, Sören Möller, Henrik Daa Schrøder, Eva Kildall Hejbøl, Abdelkhalek Samy Abdelkhalek, Niels Bjørn, Súsanna Ákusdóttir Peterson, Kristine Cederstrom Larsen, Erik Omling, Martin Salö, Oliver J. Muensterer, Mark Bremholm Ellebaek

**Affiliations:** 1 Paediatrics, Department of Clinical Sciences Lund, Lund University, Lund, Sweden; 2 Research Unit of Urology, Department of Clinical Research, University of Southern Denmark, Odense, Denmark; 3 Department of Urology, Odense University Hospital, Odense, Denmark; 4 Pediatric surgery, Women’s and Children’s Health, Uppsala University, Uppsala, Sweden; 5 Centre of Excellence in Gastrointestinal Diseases and Malformations in Infancy and Childhood (GAIN), Odense University Hospital, Odense, Denmark; 6 Research Unit for Surgery, Department of Clinical Research, University of Southern Denmark, Odense, Denmark; 7 Epidemiology, Biostatistics and Biodemography, Department of Public Health, University of Southern Denmark, Odense, Denmark; 8 Research Unit of Pathology, Department of Clinical Research, University of Southern Denmark, Odense, Denmark; 9 Winsløw Unit for Anatomy, Histology and Plastination, Department of Molecular Medicine, University of Southern Denmark, Odense, Denmark; 10 Harper & Keele Veterinary School, Keele University, Keele, England; 11 Department of Paediatric and Adolescent Surgery, Skåne University Hospital, Lund, Sweden; 12 Department of Pediatric Surgery, Dr. von Hauner Children’s Hospital, Munich, Germany; 13 Ludwig-Maximilians-Universität München, Munich, Germany

**Keywords:** Esophageal atresia, Botulinum toxin type A, Anastomotic stricture, Anastomotic leakage, Pediatric surgery, Animal trial, Diseases, Gastroenterology, Medical research

## Abstract

Anastomotic strictures and leaks are severe complications following esophageal atresia (EA) repair. We hypothesized that esophageal injections of botulinum toxin type A (BTX-A) could mitigate these complications through muscle relaxation. This randomized, controlled, blinded animal trial evaluated the feasibility and safety of a long- and short-gap porcine EA model. Furthermore, the effect of BTX-A as a surgical adjunct on anastomotic outcomes were exploratory assessed. Twenty-four animals received BTX-A or saline pre- or intraoperative to a three- or one-centimeter esophageal resection and primary anastomosis. The primary feasibility endpoint was animal completion rate at postoperative day 14, while stricture severity was the primary exploratory outcome. Secondary exploratory outcomes were anastomotic leakage frequency, and biomechanical and histological characteristics. Animal completion rate was 62.5% (15/24). There was no difference in stricture severity between the intervention and control groups (Esophageal Anastomotic Stricture Index 0.41 [IQR 0.26–0.55] versus 0.35 [IQR 0.27–0.58]; *p* = 0.73). One animal in the control group suffered an anastomotic leak. Biomechanical properties and anastomotic healing did not differ. While the procedure was technically feasible, refinement is necessary to reduce attrition rate and establish a safe model for long-term evaluation. More studies are needed to determine whether BTX-A can provide a reduction in anastomotic complications.

## Introduction

### Esophageal atresia

Esophageal atresia (EA) is the most common malformation of the esophagus with a birth prevalence of approximately 1:4000^1–3^. While the embryogenesis of EA remains poorly understood, an abnormal development of the embryonic foregut results in an incomplete fusion of the upper and lower esophageal pouches^4^. Most affected children undergo definitive surgical repair with a primary anastomosis of the esophageal pouches and ligation of the tracheoesophageal fistula within the first days of life^5^. In 10–15% of children a long-gap EA (LGEA), commonly defined as a distance between the two pouches exceeding 2.5–3 cm or more than three to four vertebral bodies, is present^6^. In these cases, a primary anastomosis is often not feasible or will result in a very high tension on the anastomosis^7^. The optimal management of these children remains debated and current techniques include organ interposition, traction procedures, and awaiting spontaneous growth of the pouches to perform a delayed anastomosis^8–12^.

While the overall mortality has decreased substantially during the last decades, morbidity and the need for reinterventions for patients with corrected EA remains considerably high^13,14^. The most common and serious postoperative complications include anastomotic strictures, defined as a narrowing of the lumen at the level of the anastomosis, and anastomotic leak^15–18^. The risk for these complications increases with the tension on the tissue, such as in cases of delayed repair in LGEA^19–22^.

### Botulinum toxin type A

In previous animal studies we have demonstrated that local treatment with the neurotoxin botulinum toxin type A (BTX-A), commonly known as Botox, significantly increases esophageal elongation and elasticity^23^. We have also shown that the effect is dosage- and time-dependent and that endoscopic administration is safe and feasible^24–26^. Studies in small animals further suggest that BTX-A treatment can reduce stricture formation following esophageal anastomosis^27,28^.

The metalloprotease BTX-A is produced by the anaerobic gram-positive bacteria *Clostridium botulinum*^29^. It targets the neuromuscular junction, where it binds to the presynaptic membrane and is internalized via endocytosis^30^. It acts by selectively cleaving the synaptosomal-associated protein (SNAP)-25. This blocks the vesicles containing acetylcholine from fusing with the presynaptic membrane, causing a transient muscle paralysis in both striated and smooth muscles^30,31^. BTX-A also inhibits the release of other neurotransmitters important for tonic muscular status, including adenosine triphosphate^32^.

By achieving esophageal muscle relaxation, tension-related anastomotic complications, including strictures and leaks, could be mitigated. Ultimately, tissue relaxation may enable a primary anastomosis in children with LGEA, with the potential to reduce short- and long-term complications, shorten hospital stays, and improve functional results. However, despite its clinical potential, it is not known how BTX-A affects tension-related anastomotic outcomes in a large-animal model after a longer postoperative period. Furthermore, there is a lack of evidence on whether the treatment has any detrimental or beneficial effect on the biomechanical aspects of the tissue, or on anastomotic healing.

### Aims and objectives

The overall aim of this study was twofold. Given that no prior studies have assessed the effect of BTX-A as an adjunct to EA repair in a large-animal model with an extended follow-up, this study aimed to evaluate the feasibility and safety of the proposed study protocol. In parallel, we aimed to exploratorily evaluate how esophageal BTX-A injections affect postoperative anastomotic characteristics in a porcine model of short- and long-gap esophageal reconstruction.

## Methods

The study was conducted at the University of Southern Denmark as part of a European collaboration with Ludwig-Maximilians-University Munich (Germany) and Lund University (Sweden). The study protocol has been published, and a pilot study involving four animals was conducted prior to this trial^33^. The study was performed in compliance with the ARRIVE guidelines for animal studies^34^.

### Study design

**Fig. 1:**
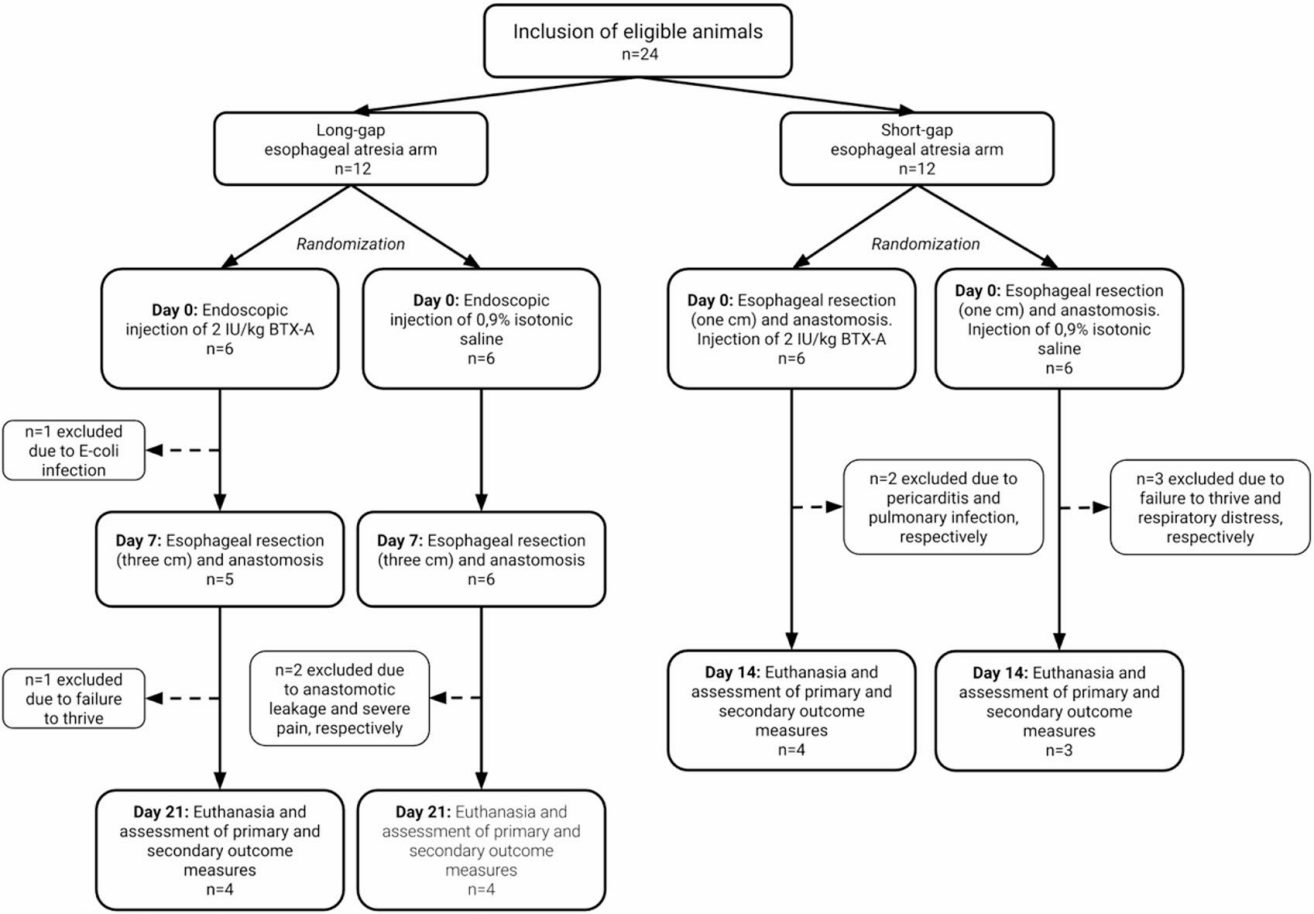
Overview of the study design, including time points and reasons for animal exclusion. Procedural steps for the long-gap esophageal atresia arm are presented in the two left columns, and for the short-gap arm in the two right columns. BTX-A=botulinum toxin type A. IU=international units.

This was a randomized, controlled, investigator-blinded animal trial. Twenty-four naïve piglets of Danish landrace x Yorkshire breed, sourced from a local multiplication herd (Kokkenborg ApS, DK-5771 Stenstrup) with the highest possible health status according to the Danish Specific Pathogen Free Health Program (Red SPF), were included at an age of seven to eight weeks, with a weight of approximately 15 kg at surgery. Animals were equally divided into a LGEA and short-gap EA (SGEA) arm. Within each arm, animals were randomized 1:1 to the intervention group receiving BTX-A, or the control group receiving isotonic saline. Animals in the LGEA arm were given injections endoscopically seven days prior to esophageal resection and anastomosis. To adhere to current clinical practice in the surgical management of children with EA, animals in the SGEA arm received injections intraoperative. All animals were re-anesthetized on postoperative day 14 and assessed for outcome measures. The study design is detailed in Fig. 1.

### Sample size estimation

A sample size calculation was conducted for the primary exploratory outcome. Usui et al. assessed postoperative esophageal anastomotic stricture formation in rabbits following BTX-A treatment^28^. In the intervention group, the mean Esophageal Anastomotic Stricture Index (EASI) was 0.446, compared to a mean EASI of 0.303 in the control group. Based on these results, and assuming a significance level of 0.05, a power of 80%, and a standard deviation of 0.1, a total of nine animals had to be included in each group. By including 24 pigs, a power of 92% comparing the intervention and the control groups was achieved. This allowed for a drop-out of 10–15% while ensuring a power of at least 80%. Including 24 animals further allowed a power of 61% for subgroup comparisons within the LGEA and SGEA arms, respectively.

### Randomization and blinding

A simple randomization strategy was applied^35^. Sealed envelopes containing a letter stating group allocation (intervention or control group) were prepared for the LGEA and SGEA arms separately. Prior to the first procedure, an envelope was randomly drawn and opened, and the group allocation was noted. The BTX-A and isotonic saline solutions were prepared in identical syringes containing the same amount of fluid before they were delivered to the operating room, to preclude any knowledge of group allocation during the intervention. The randomization was conducted and recorded by a research group participant with no involvement during the surgical procedures or data analysis. Group allocation was disclosed upon completion of data analysis.

### Pharmacological intervention and dosing

In the intervention group, animals were given two international units (IU) per kg body weight of BTX-A solution (Xeomin^®^, Merz Pharmaceuticals GmbH, Frankfurt/Main, Germany). This dose was selected as prior data showed no additional effect with higher doses; increasing the dosage to four IU per kg yielded no further gain in bursting pressure^24^. Furthermore, a dose of four IU per kg was associated with severe adverse events and high attrition rates in a previous rodent model^27^. In the control group, animals received 0.9% isotonic saline. All animals received a total of 1.2 ml of the study solution, administered via twelve injections of 0.1 ml each.

### Procedure description

**Fig. 2:**
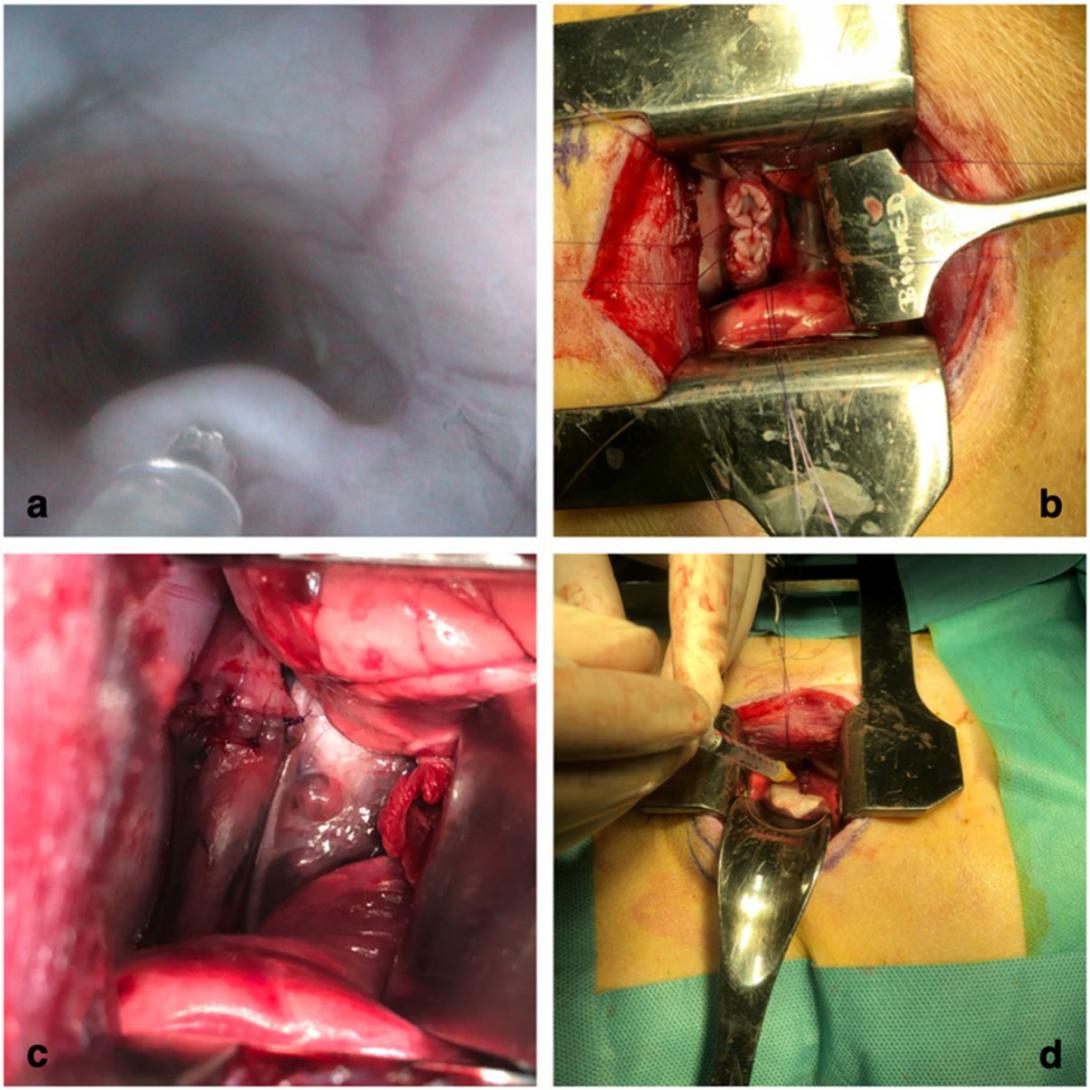
Images from the endoscopic and surgical procedures. **a**: Endoscopic esophageal intramuscular injection of the study solution in the long-gap esophageal atresia arm. **b**: Esophageal resection is completed and the anastomosis of the proximal and distal esophagus is ongoing. **c**: Completed anastomosis with full-wall interrupted, synthetic, absorbable 4–0 sutures. Ink from endoscopic tattoos can be seen in the esophageal tissue and surrounding lymph nodes. **d**: Injection of study solution in the short-gap esophageal atresia arm following completion of the anastomosis.

In the LGEA arm, animals were anesthetized and underwent an esophagoscopy using a 7.8-mm gastroscope (Storz^®^, Tuttlingen, Germany). A five-cm segment of the mid-to-distal thoracic esophagus was marked using endoscopic tattooing (GI SPOT^®^, Braun Scandinavia A/S, Værløse, Denmark). The study solution was injected into the esophageal muscle layer at three levels distally and proximally to the marked segment, with two depots at each level. After seven days, a right-sided posterolateral thoracotomy was performed. A three-cm-long esophageal resection was conducted between the tattoos, followed by an end-to-end anastomosis using interrupted synthetic absorbable monofilament 4–0 sutures. In the SGEA arm, a one-cm-long esophageal resection with an end-to-end anastomosis was performed. Upon completion of the anastomosis, the study solution was injected at three levels distally and proximally to the anastomosis with two depots at each level. The different procedural steps are illustrated in Fig. 2. The anesthetic procedure and the protocol for intra- and postoperative pain management and monitoring are detailed in the study protocol^33^.

### Primary outcomes

Given the dual role as a feasibility and exploratory study, the primary outcomes were categorized accordingly. To evaluate the feasibility of the model, the animal completion rate, defined as the proportion of animals reaching the prespecified endpoint at postoperative day 14, constituted the first primary outcome. The exploratory primary outcome was anastomotic stricture severity, assessed on postoperative day 14. Stricture severity was calculated using the EASI, defined as:

$$\:\mathrm{E}\mathrm{A}\mathrm{S}\mathrm{I}\:=\frac{\mathrm{l}\mathrm{a}\mathrm{t}\mathrm{e}\mathrm{r}\mathrm{a}\mathrm{l}\frac{d}{D}+\mathrm{a}\mathrm{n}\mathrm{t}\mathrm{e}\mathrm{r}\mathrm{o}\mathrm{p}\mathrm{o}\mathrm{s}\mathrm{t}\mathrm{e}\mathrm{r}\mathrm{i}\mathrm{o}\mathrm{r}\frac{d}{D}}{2}$$

As an additional measure of the stricture degree the Anastomotic Index was calculated, using the following Eq. 3^6^:

$$\:\mathrm{A}\mathrm{a}\mathrm{n}\mathrm{a}\mathrm{s}\mathrm{t}\mathrm{o}\mathrm{m}\mathrm{o}\mathrm{t}\mathrm{i}\mathrm{c}\:\mathrm{I}\mathrm{n}\mathrm{d}\mathrm{e}\mathrm{x}\:=\frac{2\:\mathrm{x}\:d}{\mathrm{m}\mathrm{e}\mathrm{a}\mathrm{n}\:\mathrm{p}\mathrm{r}\mathrm{o}\mathrm{x}\mathrm{i}\mathrm{m}\mathrm{a}\mathrm{l}\:D\:+\:\mathrm{m}\mathrm{e}\mathrm{a}\mathrm{n}\:\mathrm{d}\mathrm{i}\mathrm{s}\mathrm{t}\mathrm{a}\mathrm{l}\:D}$$

In both calculations, *d* is the anastomotic diameter, and *D* is the proximal or distal pouch diameter on a contrast radiograph^37^. Pouch diameter was averaged from measurements at three levels above and below the anastomosis to determine proximal and distal *D*. Hence, calculating the EASI generates two estimates (an upper and lower-EASI, respectively). As lower-EASI best predicts the need for at least one stricture dilation, results were restricted to only include this measurement^37^.

To evaluate the stricture severity, in and ex vivo contrast radiographs were obtained. With the animal in general anesthesia, an 18 Charrière Foley catheter was inserted orally with the tip placed approximately ten cm proximal to the anastomosis. The catheter balloon was inflated to occlude the lumen. Omnipaque^®^ 40 ml contrast medium (140 mg/ml of lohexol, GE Healthcare, Brøndby, Denmark) was injected and a dynamic contrast esophagogram was conducted. The esophagus was surgically removed via a right-sided thoracotomy and clamped proximally and distally to the anastomosis. Following cannulation and infusion of contrast medium to a standardized intraluminal pressure of 20 mmHg, lateral and anteroposterior radiographs were obtained. EASI and Anastomotic Index were calculated based on these in and ex vivo radiographs, using ImageJ 4 (Rasband, W.S., ImageJ, U.S. National Institutes of Health, Bethesda, USA)^37,38^. A second examiner repeated a selection of measurements to confirm accuracy. Results were deemed reliable and the original measurements used if the intraclass correlation reached ≥ 0.9.

Indirect stricture markers were reported and included visual appearance of the anastomosis, food residuum or dilation of the upper pouch, weight gain, upper pouch diameter, and anastomotic diameter. Prior to the contrast studies, an esophagoscopy was conducted. The macroscopic appearance of the anastomosis was graded by two independent examiners on a four-digit scale, with zero being no narrowing of the anastomosis and three being a significant stricture. Weight was measured daily throughout the study. The absolute and relative weight gain until end of study was calculated. The anastomotic and mean luminal diameter proximal to the anastomosis were measured on the radiographs and calculated as previously described.

### Secondary outcomes

Secondary feasibility endpoints included animal completion rate at postoperative day ten, and the number of animals for which the primary exploratory outcome could be successfully evaluated. Furthermore, postoperative complications were reported. As animals were not under continuous 24-hour observation, no quantitative comparison of complication frequency was performed; instead, all observed complications were qualitatively described. Lastly, technical success of the surgical procedures was evaluated to identify areas for methodological improvement.

Secondary exploratory outcomes included anastomotic leak frequency, intraluminal anastomotic pressure, esophageal elongation and anastomotic strength, and healing at the site of the anastomosis. Anastomotic leak was defined as an effusion of contrast medium on the in vivo contrast study or as a visible insufficiency upon macroscopic inspection of the specimen. To assess the anastomotic pressure, a Uni-Tip Catheter (UniSensor AG, Attikon, Switzerland) connected to a Medical Measurement Urodynamic system (MMS, Enschede, Netherlands) was inserted orally into the stomach. It was withdrawn through the esophagus at a constant speed of five mm/s. The baseline and anastomotic pressures were recorded, and the pressure difference was calculated.

Esophageal elongation and anastomotic strength were assessed using a dynamic stretch-tension test after 25 min of cold ischemia. The specimen was mounted in a stretch-tension device (LFPlus Series Universal Test Machine, Lloyd Instruments LTD, Hampshire, UK) with three cm between the clamps and the anastomosis placed in the center. To compensate for any length or tension differences a preload of two newtons (N) was applied, and the specimen was stretched at a constant rate of 30 mm per minute until transmural tear appeared. Elongation from preload, maximum load, and the tensile strength to cause rupture, confirmed by a drop in the load-strain curve calculated by the software, was recorded.

Lastly, anastomotic healing was assessed histologically. The specimen including the anastomosis was fixed in buffered formalin and embedded in paraffin. Sections of two µm were cut and stained on BenchMark, Ventana or Omnis, Dako Agilent, instruments. Muscle regeneration was assessed by immunohistochemistry for myoD, myogenin, and paired box 7 (PAX7). Desmin was used for detection of muscle. CD68 and T- and B-cell markers were applied for detection of macrophages and lymphocytes, respectively. Assessment of fibrosis degree using Sirius Red staining was attempted; however, due to the mechanical testing conducted prior to fixation, a formal quantitative comparative analysis was not feasible. By conducting an immunohistochemical analysis for synaptophysin, the presence and density of ganglion and nerve terminals proximal and distal to the anastomosis was assessed.

### Statistical analysis

Continuous variables were reported as median and interquartile range (IQR). Differences between groups were analyzed using a Wilcoxon rank-sum test for continuous variables and Fisher’s exact test for categorical variables. For all outcomes, subgroup analyses were conducted for the LGEA and SGEA arms separately. Notably, secondary outcomes and subgroup analyses were not formally powered to detect statistically significant differences between groups. Furthermore, given the limited sample size, findings from these comparisons should not be considered confirmatory but interpreted as strictly hypothesis-generating. For the primary exploratory outcome, a two-sided p-value of < 0.05 was considered statistically significant. Statistical analysis was conducted using Stata (StataCorp. 2019. Stata Statistical Software: Release 16.1. College Station, TX: StataCorp LLC).

### Deviations from the study protocol

To ensure full transparency, we wish to acknowledge the modifications made to the previously published study protocol^33^. While the original protocol focused exclusively on the efficacy of BTX-A regarding anastomotic stricture severity, leak rates, and mechanical and histological tissue properties, the high complexity of the model necessitated a refined focus. Consequently, the study’s scope was expanded to include a dual primary aim of evaluating the study’s feasibility. To address this, we integrated predefined feasibility endpoints, including animal completion rate, procedural success, and postoperative morbidity. Accordingly, the outcomes originally defined as primary have been reclassified as exploratory. These modifications were implemented to provide a more rigorous and balanced evaluation of the porcine model and to offer critical insights for future methodological refinements. Furthermore, while the original protocol suggested a regression model to adjust the primary outcome for weight at the end of the study, this was subsequently omitted. Due to the high attrition rate, the resulting sample size lacked the statistical power required for a meaningful multivariate analysis.

### Ethics declarations

The study was approved by the Danish Animal Experimentation Council (application number 2020-15-0201-00512) and was conducted in accordance with relevant guidelines and regulations. Animals were stabled together with access to heating lamps, softened food, and water, except from the first 48 h postoperative when each animal was housed in a separate stall. Body temperature and weight was registered once daily. All animals were assessed every sixth to eight hours during the first 48 h following the procedures, and twice daily in the remaining time. If an animal showed signs of distress, such as inappetence, inactivity, increased heart or respiratory rate and temperature, diarrhea, drooling or vomiting, teeth grinding or hanging tail, or failure to thrive, the surveillance was intensified, and a veterinarian was alerted to assess the animal. In case of severe infection, pain that could not be treated with analgesics, or failure to thrive, animals were euthanized in accordance with the pre-stated humane endpoints described in the study protocol^33^. Animals were euthanized at the end of the final procedure, directly following harvesting of the esophagus. Animals received an intravenous dose of pentobarbital 140 mg/kg, a short-acting barbiturate commonly used and accepted for euthanasia in animals, while still under general anesthesia^39^.

## Results

A total of 24 pigs were included: 18 (75%) boars and six (25%) sows. Apart from mean intraoperative arterial pressure and body temperature, which were higher in the control group (72 mmHg [IQR 69–77] versus 85 mmHg [IQR 83–91]; *p* = 0.03, and 37.5^°^C [IQR 37.1–37.9] versus 38.1^°^C [IQR 37.5–38.3]; *p* = 0.03), pre- and intraoperative measures were similar between groups and are presented in Table 1.

**Table 1:** Weight at inclusion and intraoperative measures for animals surviving 14 days following esophageal resection and anastomosis.

	Intervention group (*n *= 8)	Control group (*n *= 7)	*p*-value
Weight at inclusion (kg)	15.5 (14.7-15.9)	15 (14-16.2)	0.82
Length of surgery (minutes)	113 (105-128)	140 (110-150)	0.27
Mean arterial pressure (mmHg)	72 (69-77)	85 (83-91)	0.03
Heart rate (beats per minute)	90 (86-95)	94 (92-109)	0.09
Body temperature (C°)	37.5 (37.1-37.9)	38.1 (37.5-38.3)	0.03
Oxygen saturation (%)	98 (98-99)	99 (98-100)	0.18

### Animal completion rate, morbidity, and reasons for exclusion

The median survival after surgery was 14 days in both the intervention and control groups (IQR 13–14 versus 8–14; *p* = 0.49). Fifteen animals (62.5%) completed the study: eight (67%) in the intervention and seven (58%) in the control group (odds ratio 0.7; 95% confidence interval 0.13–3.68). Nine animals in each group (9/12; 75%), survived ten days or longer (odds ratio 1; 95% CI 0.16–6.35). One animal in the intervention group was euthanized after the endoscopic procedure but prior to surgery due to a confirmed *Escherichia coli* infection causing severe diarrhea and failure to thrive. Three more animals in the intervention group were euthanized two, six and 13 days postoperative due to failure to thrive, apathy, and respiratory distress, respectively. In the first case, no macroscopic abnormality was found. In the second case, autopsy revealed an enlarged heart with fibrin coating and pericardiac fluid, indicative of pericarditis. In the third case, the animal had a pleural empyema but no sign of anastomotic leak on the contrast radiograph or upon inspection of the specimen.

In the control group, five animals were euthanized on postoperative day three, four, six, and ten (*n* = 2), respectively. In the first case, the animal presented with dyspnea, failure to thrive, and progressive subcutaneous emphysema. Anastomotic leakage was confirmed on autopsy. Among the other four animals, two had macroscopic findings consistent with pulmonary infection and pericarditis, while two had no abnormal findings on autopsy. In the control group, five (5/7; 71%) of the animals surviving until the end of study suffered from late postoperative emesis, whilst four (4/8; 50%) of animals in the intervention group showed similar symptoms. Other postoperative complications observed included local swelling and surgical site infections, decreased food intake, transient respiratory distress, and tachycardia. In five animals, one in the intervention group and four in the control group, transient muscle hypotonia was observed following surgery.

### Anastomotic stricture severity

The primary exploratory outcome, anastomotic stricture severity, could be assessed in all animals completing the study. The lower-EASI was 0.29 [IQR 0.24–0.4] in the intervention group and 0.32 [IQR 0.2–0.36] in the control group (*p* = 0.56). Neither the Anastomotic Index nor the anastomotic diameter differed between the groups (0.26 [IQR 0.2–0.37] versus 0.23 [IQR 0.14–0.32]; *p* = 0.36, and 0.51 cm [IQR 0.37–0.64] versus 0.36 cm [IQR 0.34–0.63]; *p* = 0.56). The relative weight gain was similar between the groups (59% [IQR 35.5–79.5] versus 41.6% [IQR 32.7–57.3]; *p* = 0.36). The intraclass correlation for the contrast radiograph measurements was 0.95 (95% CI 0.74–0.99). All results on direct and indirect stricture severity are outlined in Tables 2 and 3, respectively.

**Table 2:** Measures of anastomotic stricture severity from in and ex vivo contrast studies at 14 days postoperative to esophageal resection and anastomosis.

	Intervention group (*n* = 8)	Control group (*n* = 7)	*p*-value
Lower-EASI, in vivo	0.41 (0.26-0.55)	0.35 (0.27-0.58)	0.73
*LGEA*	0.41 (0.25–0.52)	0.33 (0.24–0.37)	0.56
*SGEA*	0.42 (0.26–0.57)	0.58 (0.27–0.61)	0.16
Lower-EASI, ex vivo	0.29 (0.24–0.40)	0.32 (0.2–0.36)	0.56
*LGEA*	0.33 (0.24–0.46)	0.26 (0.17–0.32)	0.39
*SGEA*	0.27 (0.24–0.37)	0.36 (0.23–0.38)	1
Anastomotic Index, in vivo	0.42 (0.25–0.51)	0.3 (0.23–0.47)	0.3
*LGEA*	0.42 (0.26–0.51)	0.3 (0.21–0.31)	0.15
*SGEA*	0.39 (0.25–0.53)	0.47 (0.23–0.51)	0.48
Anastomotic Index, ex vivo	0.26 (0.2–0.37)	0.23 (0.14–0.32)	0.36
*LGEA*	0.31 (0.21–0.4)	0.18 (0.13–0.25)	0.15
*SGEA*	0.23 (0.2–0.32)	0.32 (0.2–0.33)	0.72

Wilcoxon rank-sum test. Results presented as median (interquartile range) for all animals, and for the LGEA (*n* = 8) and SGEA (*n* = 7) arms separately. EASI=esophageal anastomotic stricture index. LGEA=long-gap esophageal atresia. SGEA=short-gap esophageal atresia.

**Table 3:** Indirect measures of anastomotic stricture severity from contrast studies and esophagoscopy 14 days postoperative to esophageal resection and anastomosis.

	Intervention group (*n* = 8)	Control group (*n* = 7)	*p*-value
Weight at euthanasia (kg)	24.4 (20.7-26.8)	22.3 (20.5-22.8)	0.1
*LGEA*	26.8 (24.9–30)	21.7 (20.7–23.6)	0.04
*SGEA*	20.7 (19.1–22.9)	22.3 (18.6–22.8)	0.72
Weight gain (kg)	9.1 (5.2–11.8)	6.5 (5.5–8.2)	0.42
*LGEA*	11.8 (9.7–15)	7.4 (6.2–9.9)	0.08
*SGEA*	5.2 (3.9-7)	5.5 (2.4–6.7)	1
Relative weight gain (%)	59 (35.5–79.5)	41.6 (32.7–57.3)	0.36
*LGEA*	79.5 (63.8–99.5)	51.9 (42.9–72.6)	0.08
*SGEA*	35.5 (24.3–46.5)	32.7 (14.8–41.6)	0.48
Anastomotic diameter (cm)	0.51 (0.37–0.64)	0.36 (0.34–0.63)	0.56
*LGEA*	0.45 (0.3–0.63)	0.35 (0.31–0.4)	0.56
*SGEA*	0.52 (0.43–0.68)	0.63 (0.36–0.81)	0.72
Upper pouch diameter (cm)	1.43 (1.16–1.49)	1.65 (1.27–1.82)	0.11
*LGEA*	1.25 (1.02–1.43)	1.46 (1.27–2.01)	0.25
*SGEA*	1.49 (1.36–1.80)	1.76 (1.65–1.82)	0.29
Endoscopic anastomotic stricture^1,2^			0.18
*None*	0 (0)	0 (0)	
*Slight*	3 (37.5)	0 (0)	
*Moderate*	3 (37.5)	2 (28.6)	
*Pronounced*	2 (25)	5 (71.4)	
Endoscopic upper pouch dilation^1,3^	1 (12.5)	3 (42.9)	0.28

Wilcoxon rank-sum test and Fisher’s exact test. Results presented as median (interquartile range) for all animals, and for the LGEA (*n* = 8) and SGEA (*n* = 7) arms separately, unless indicated otherwise. ^1^Results presented as n (%) of the total cohort. ^2^Kappa value 0.33 (standard error 0.16). ^3^Kappa value 0.41 (standard error 0.25). LGEA=long-gap esophageal atresia. SGEA=short-gap esophageal atresia.

### Anastomotic leak, intraluminal pressure at anastomosis, and esophageal elongation

One animal in the control group, and none in the intervention group, developed an anastomotic leak. The intraluminal pressure at the anastomosis was 24 cmH_2_O [IQR 13-26.5] in the intervention group and 17.5 cmH_2_O [IQR 17.5–23] in the control group (*p* = 0.81). The relative tissue elongation was slightly increased in the intervention group (85% [IQR 71–93] versus 78% [IQR 71–80]; *p* = 0.25). The maximum load to cause transmural tissue rupture was slightly lower in the intervention group (51.7 N [IQR 46-64.6] versus 70.5 N [IQR 65.4–75]; *p* = 0.06). All results are outlined in Table 4.

**Table 4:** Anastomotic pressure profile and esophageal elongation and elasticity at 14 days postoperative to esophageal resection and anastomosis.

	Intervention group (*n* = 8)	Control group (*n* = 7)	*p*-value
Intraluminal pressure (cmH_2_O)^1^	13.5 (12-15)	15.5 (13.5-16)	0.19
*LGEA*	12.5 (12–15)	14.8 (13.3–17.8)	0.16
*SGEA*	14 (12.5–15.5)	15.5 (15.5–15.5)	0.48
Anastomotic pressure (cmH_2_O)^1^	37.5 (26.5–40)	33.5 (31-42.5)	0.81
*LGEA*	38.5 (26.5–40)	32.2 (28.5–38)	0.72
*SGEA*	32.5 (26-52.5)	55 (55–55)	0.48
Pressure difference (cmH_2_O)^1^	24 (13-26.5)	17.5 (17.5–23)	0.81
*LGEA*	25 (14-26.5)	17.5 (15.3–20.3)	0.29
*SGEA*	18.5 (13-37.5)	39.5 (39.5–39.5)	0.47
Elongation from preload (mm)^2^	13.8 (10.1–15.7)	14.6 (13.4–16.3)	0.89
*LGEA*	14 (10.1–18.5)	16.3 (15.8–17.2)	0.51
*SGEA*	13.7 (11-14.7)	13.4 (7.7–13.5)	0.16
Relative elongation (%)^2^	85 (71–93)	78 (71–80)	0.25
*LGEA*	93 (85–95)	80 (71–89)	0.13
*SGEA*	77 (62–89)	76 (59–80)	0.72
Load to cause rupture (Newton)^2^	51.7 (46-64.6)	70.5 (65.4–75)	0.06
*LGEA*	64.6 (46.9–81)	70.8 (65.4–84.3)	0.28
*SGEA*	48.8 (44.1–55.1)	70.2 (48.5–75)	0.16

Wilcoxon rank-sum test. Results presented as median (interquartile range) for all animals, and for the LGEA (*n* = 8) and SGEA (*n* = 7) arms separately. ^1^Data available for twelve animals. ^2^Data available for 13 animals. BTX-A=botulinum toxin type A. LGEA=long-gap esophageal atresia. SGEA=short-gap esophageal atresia.

### Histology

**Fig. 3:**
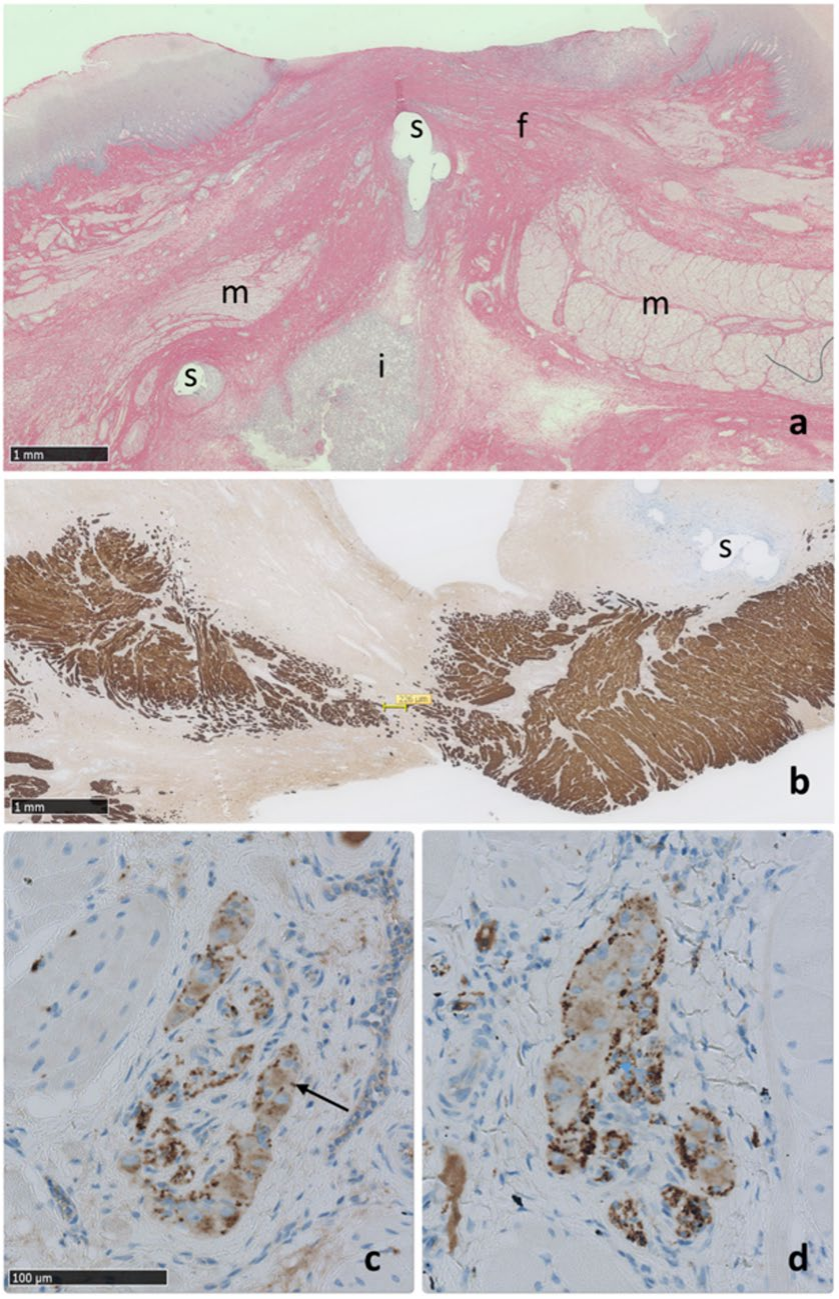
Sections displaying morphology at the esophageal anastomosis in the specimen at postoperative day 14. **a**: Sirius Red stain demonstrating the fibrotic reaction (f), inflammation (i), and the suture (s). Note: formal quantification of fibrosis was precluded by structural alterations from preceding mechanical testing. The distance between the muscle (m) elements at the anastomosis is 2.1 mm. **b**: Desmin stain highlighting skeletal muscle. The distance between the muscle elements is 226 $\:\mu\:$m. **c **and **d**: Synaptophysin stain showing a ganglion above (c) and below (d) the anastomosis. Nerve terminals (indicated by black arrow in Fig. 3c) are present in both locations.

The distance between the muscle fibers at the site of the anastomosis was 1.9 mm [IQR 1.1–4.8] in the intervention group and 3.1 mm [IQR 2.5–5.5] in the control group (*p* = 0.39). In all specimens, ganglions and nerve terminals were present in the muscular plexus (Fig. 3). Due to the preceding mechanical testing, the fibrosis degree could not be determined. Furthermore, the degree of inflammation varied largely within each specimen, making comparisons between the groups unreliable.

### LGEA subgroup analysis

In the LGEA arm, the weight at the end of study was 26.8 kg [IQR 24.9–30] in the intervention group, and 21.7 kg [IQR 20.7–23.6] in the control group (*p* = 0.04). The relative weight gain was 79.5% [IQR 63.8–99.5] and 51.9% [IQR 42.9–72.6] (*p* = 0.08). The anastomotic diameter was 0.45 cm [IQR 0.3–0.63] in the intervention group, compared to 0.35 cm [IQR 0.31–0.4] in the control group (*p* = 0.56), while the upper pouch diameter was 1.25 cm [IQR 1.02–1.43] and 1.46 cm [IQR 1.27–2.01], respectively (*p* = 0.25). The relative tissue elongation was 93% [IQR 85–95], in the intervention group, compared to 80% [IQR 71–89] in the control group (*p* = 0.13), while the distance between muscle fibers was 1.6 mm [IQR 1-1.9] and 2.9 mm [IQR 1.6–3.3] (*p* = 0.19), respectively (Tables 2, 3 and 4).

## Discussion

Complications following surgical repair of both short- and long-gap EA remain a clinical challenge. In this randomized, controlled, blinded animal trial, we sought to evaluate the feasibility and safety of the proposed porcine model, while exploratory assessing the effects of perioperative BTX-A injections on the most common and severe postoperative complications and underlying mechanical risk factors.

While we could evaluate stricture severity in all animals, we could not see any significant difference between the intervention and the control groups. Indirect measures of distal esophageal obstruction, such as poor weight gain, anastomotic diameter, and upper pouch diameter, were similar between groups. Although these measures are suggested to predict the need for stricture dilations, it is important to note that radiological stricture degree does not always correlate with clinical symptoms and thus the need for treatment^15,22^. While the nature of this study precluded evaluation of symptom severity, indirect signs of obstruction, such as regurgitation and failure to thrive, were recorded in both groups.

The pathogenesis of anastomotic strictures remains incompletely understood, but factors such as leaks and local ischemia, often exacerbated by poor healing or high tension on the tissue, increase the risk^21,40,41^. Contrary to prior evidence, the current study did not demonstrate any pronounced increase in tissue elongation following BTX-A treatment^23,24,26^. However, and in line with previous reports, the load required to cause transmural rupture was generally lower in the intervention group^24^. This observation has previously been interpreted as a sign of increased tissue compliance induced by BTX-A, contrasted against the relative rigidity and stiffness of the control group requiring an increased load to cause tissue disruption. However, it cannot be ruled out that this finding reflects a decrease in structural strength which might negatively impact the anastomosis. While being exploratory results that should be interpreted with caution, further studies are required to determine whether this shift represents a beneficial increase in pliability or a clinically relevant weakening of the anastomotic site.

Prior studies have raised concerns regarding BTX-A-related complications. While the toxin can spread to adjacent muscles by a concentration gradient, systemic effects through hematogenous spread or retrograde axonal transport are rare^42^, and autonomic side effects seem exceedingly limited^43^. BTX-A is used for various esophageal conditions in both adults and children with no evidence of systemic toxicity; its safety profile is supported by several studies reporting only minor, transient local effects^44–48^. Despite this evidence, a previous rodent study reported several animals with signs of severe systemic toxicity^27^. No such findings were observed in our cohort. While some animals showed signs of systemic inflammation, there was no difference in the number or severity of complications between the intervention and control groups. We suspect that these symptoms stemmed from the surgical trauma or pre-existing conditions, rather than the toxin itself. However, when considering these findings, it should be acknowledged that some intraoperative parameters, including mean arterial pressure and body temperature, differed between groups. These differences were minor in absolute terms and remained only marginally below normal physiological ranges (median body temperature was 37.5 °C in the intervention group and 38.1 °C in the control group). They are therefore unlikely to be of clinical relevance and did not appear to influence postoperative outcomes or the overall safety profile of the intervention.

Beyond immediate safety, long-term esophageal hypo- and dysmotility following EA repair has remained a concern^49^. While the paralytic effect of BTX-A is potent, it is inherently transient, typically declining over three to four months in skeletal muscle and up to one year in autonomic nerve terminals^50^. Muscle regeneration at the anastomotic site appeared unaffected with no pronounced difference in the distance between muscle fibers, indicating that the toxin selectively modulates muscle tone without compromising the underlying tissue’s capacity for healing and remodeling. Furthermore, in the histological assessment, intact ganglion cells and nerve terminals were identified in all animals. While these results are promising and alleviate concerns raised by previous reports of BTX-A-induced neural damage^27^, it is important to note that postoperative esophageal motility and neural function were not assessed. Consequently, further studies are required to determine whether BTX-A risks exacerbating the inherent dysmotility observed in EA patients.

### Methodological considerations and limitations

The porcine esophagus exhibits distinct biomechanical and histological characteristics compared to rodents and humans, including a thicker wall and greater tensile strength, which makes it suitable for surgical modeling^51,52^. Furthermore, pigs and humans share a similar muscle distribution, with approximately two-thirds striated muscle proximally and one-third smooth muscle distally^53^. While this study demonstrated that the surgical procedures and BTX-A administration were technically feasible, with no intraoperative or immediate postoperative mortality, the proposed model requires refinement. High complication and attrition rates impacted the outcomes, and future studies must consider improving diagnostic procedures and refining critical experimental aspects to improve animal safety and overall model feasibility. Some of the key methodological considerations are elaborated below.

First, the BTX-A dose of two IU per kg, corresponding to approximately 30 IU per animal, was chosen based on conservative safety data from a rodent model^27^. Considering pediatric clinical practice, where doses of 100–200 IU are common without serious side effects^54,55^, our dose may have been sub-therapeutic. Additional critical variables to consider for BTX-A efficacy are injection technique and timing of administration in relation to surgery. While the evidence is conflicting, reducing the number of injection sites to increase concentrations or targeting the motor endplate region may enhance the local effect^27,56^.

More consistently, while esophageal elongation increases after only two hours from administration^26^, the full effect of BTX-A is reached after one to two weeks^50^. Hence, the differences in anastomotic outcomes were expected to be more pronounced in the LGEA arm, where BTX-A was administered seven days prior to conducting the anastomosis. In contrast, for the SGEA arm, in which animals received intraoperative treatment to align with current clinical practice, the toxin’s ability to reduce immediate mechanical tension was likely diminished, as its paralytic effect did not reach its peak until after surgery. In a clinical setting, administrating BTX-A endoscopically, and via a gastrostomy if available, followed by a waiting period to allow for the toxin to reach its full effect, does not seem to increase short-term complications, but could avert long-term morbidity and improve functional outcomes especially for children with LGEA^57^. If a longer interval is unfeasible, case reports suggest that even a one-hour waiting period may facilitate successful delayed anastomosis in LGEA^58,59^. Regardless, future protocols should consider evaluating longer waiting intervals from injection to surgery, and higher BTX-A dosages, to achieve optimal tissue relaxation.

A considerable methodological challenge was the conflict between stricture evaluation, biomechanical testing, and histological outcome assessments. The duration of cold ischemia prior to biomechanical assessment was extended to 25 min to allow for the in and ex vivo contrast studies; in preceding studies the interval was two to seven minutes. This may have induced autolytic changes or altered the tissue’s viscoelastic properties, potentially masking the toxin’s effect. Additionally, due to the mechanical testing, the fibrosis degree or density of inflammatory markers – important indicators to determine stricture development and anastomotic healing – could not be established. To optimize future trials, we suggest separating specimens to allow for independent mechanical and histological analysis, ensuring that markers of inflammation and fibrosis can be accurately assessed^60^.

Beyond these methodological considerations, several limitations of the study necessitate discussion. While being the first study in larger animals after an extended postoperative follow-up, the evaluation period for some of the outcomes, particularly stricture formation, is relatively short. Nevertheless, it is important to note that while the median time for strictures to develop is two to three months, clinically relevant strictures can emerge as early as one to two weeks postoperatively^18,22^. Consequently, our observation window likely captures the most critical early phase of esophageal healing and remodeling.

Furthermore, the attrition rate was higher than anticipated, and the number of animals required according to the a priori sample size calculation was not met. While the original power calculation assumed an EASI difference of 0.143 (0.446 versus 0.303 in the intervention and control groups), the observed difference was only 0.06 (0.41 versus 0.35). Moreover, the observed standard deviation of approximately 0.2 was double the assumed value of 0.1. Consequently, a *post hoc* power calculation revealed a power of only 9% to detect a significant difference of the observed magnitude, and 30% for the effect size originally expected. Hence, the lack of statistical significance for the primary endpoint should be interpreted as inconclusive rather than a definitive lack of treatment effect. In this context, it should also be emphasized that the study was not powered to detect differences in secondary outcomes or to perform robust subgroup analyses, and the presented p-values should be considered strictly exploratory and descriptive rather than confirmatory.

The high attrition rate also precluded a regression analysis to adjust for body weight and other potential confounders, as such model would have been prone to overfitting. Although no causal link was established between BTX-A treatment and the complications requiring study termination, the dropout rate introduces a potential for attrition bias. These challenges underscore the necessity for careful animal selection and refined diagnostic protocols to mitigate surgical complications in future models. For instance, while anastomotic leak was only confirmed in one case, undetected contained leaks may have contributed to postoperative morbidity. To address such diagnostic challenges, future prospective studies should consider incorporating fluoroscopy to enable dynamic, real-time evaluation of anastomotic integrity^15,61^. Furthermore, employing minimally invasive surgical methods could mitigate postoperative stress and pain, which may in turn improve animal survival and study completion rates. Lastly, the inclusion of both boars and sows represents a potential source of variability. While their distribution was balanced between the intervention and control groups, future studies should consider using a genetically standardized population to minimize the impact of inherent biological variations on the study outcomes.

## Conclusion

While the model was technically feasible, the high attrition rate underscores the need for further model refinement, and a reliable assessment of safety can only be achieved after further optimization of the surgical and postoperative protocol. The study observed no differences in exploratory outcomes including anastomotic stricture severity or leak rates following BTX-A treatment. Furthermore, biomechanical and histological assessments, including esophageal elasticity, anastomotic pressure, and healing, showed only minor variations between the groups. These findings do not currently support a clinical benefit of BTX-A at the evaluated dosage. Future studies are warranted to optimize the model and should consider focusing specifically on LGEA, where the potential for reducing mechanical tension may yield a more detectable benefit.

## Data Availability

Data supporting the results presented in this article will be made available by the corresponding author on reasonable request.
